# Enabling the Circular Economy Transition in Organizations: A Moderated Mediation Model

**DOI:** 10.3390/ijerph19020677

**Published:** 2022-01-07

**Authors:** Andreea Loredana Bîrgovan, Sorin Daniel Vatca, Laura Bacali, Andrea Szilagyi, Elena Simina Lakatos, Lucian Ionel Cioca, George Ciobanu

**Affiliations:** 1 Institute for Research in Circular Economy and Environment Ernest Lupan, 400609 Cluj-Napoca, Romania; loredana.birgovan@ircem.ro (A.L.B.); laura.bacali@mis.utcluj.ro (L.B.); andrea.szilagyi@ircem.ro (A.S.); simina.lakatos@ircem.ro (E.S.L.); 2 Faculty of Industrial Engineering, Robotics and Product Management, Technical University of Cluj-Napoca, 400641 Cluj-Napoca, Romania; 3 Faculty of Agriculture, University of Agricultural Sciences and Veterinary Medicine Cluj-Napoca, 400372 Cluj-Napoca, Romania; 4 Faculty of Engineering, Lucian Blaga University of Sibiu, 550024 Sibiu, Romania; lucian.cioca@ulbsibiu.ro; 5 Academy of Romanian Scientists, 010071 Bucharest, Romania; 6 Faculty of Economics and Business Administration, University of Craiova, 200585 Craiova, Romania; george.ciobanu@edu.ucv.ro

**Keywords:** circular economy, corporate environmental responsibility, organizational change, sustainability

## Abstract

The notion of Corporate Environmental Responsibility has been extensively researched in the literature so far, but less is known about how this concept fits into the circular economy paradigm. We performed a moderated mediation analysis in order to identify the mechanism that links corporate environmental responsibility with readiness for change towards a circular economy business model. The findings from 311 respondents show that there is a positive association between corporate environmental responsibility and the readiness for change to a circular model, mediated by perceived circular economy drivers. In addition, perceived circular economy barriers hinder this positive relationship, acting as a buffer. These findings can further contribute to the elaboration of a conceptual framework for embedding circular economy in the corporate social responsibility strategies of organizations.

## 1. Introduction

The circular economy (CE) is an economic model conceived as an alternative to the linear economy with major implications for most branches of industry today. The linear model involves the production and consumption of goods or services without considering the environmental externalities (increasing waste generation, pollution, endangered biodiversity) arising from the irrational exploitation of virgin resources. Specifically, in the linear paradigm, the economic objectives tend to prevail above environmental considerations. In contrast, the circular economy refers to the production and consumption of goods and services through closed-loop material flows in which the environmental externalities are taken into account from the beginning of the design phase of the product or service. Moreover, CE accounts for the social and economic spheres at the same time (i.e., eliminate waste to prevent loss of economic value, avoid reliance on feedstocks which are subject to price fluctuations, etc.), while separating economic prosperity from the consumption of resources [1,2]. 

An essential factor contributing to the negative externalities of the linear model is the economic activity of organizations all around the world. Companies whose prosperity is inherently linked with a profligate manner of raw material consumption will have no other choice but to rethink their processes, activities and relationship with the environment. In this manner, companies can create competitive advantage and adhere to the targets imposed by the European regulations and strategies [3,4]. 

However, increased circularity in organizations means changes in the way companies understand, generate value and maintain competitive advantages. As companies are forced to interact in an ecosystem comprised of various actors, this transition requires innovation for rethinking existing business models and their collaboration approaches [5,6]. 

While scholars are focusing on identifying and understanding the relationships between innovation and organizational change, practitioners are under coercive stress to transform business models for the incorporation of circular thinking [7,8].

In order to help companies adjust economically to the new circular economy view, the concept of circular business model was developed [9,10,11]. Circular business models generally reconcile business value creation with the adoption of resource efficiency strategies, through approaches such as repair, remanufacturing or capitalizing the economic and environmental value embedded in products [5,10]. Unlike linear business models, in which a product is usually outdated after a single use and its built-in value decreases, circular business models support the development of product systems that incorporate strategies to keep the built-in value at its highest degree of utility for a long period of time [12]. 

However, until now CE has paid particular interest to waste management, decreasing the need of pristine resources and environmental impact, while overseeing business management and closing the loops into organizations. Although adopting CE is technically feasible in various cases and areas of interest, limitations come often into play during the implementation phase as economic and market constraints [4,13]. Thus, without systematic considerations of CE into the organizational strategies of companies for development, their potential for paving the transition to CE is not fully made use of.

Therefore, this present study argues that corporate environmental responsibility can lead to organizational change in terms of a transition to a circular business model, but through a mediated pathway. We conducted inferential analyzes to examine the mediating mechanism by which corporate environmental responsibility is linked to readiness for change in companies, considering the mediating influence of perceived facilitators of the CE and then the moderating role of perceived organizational barriers of the CE. Therefore, Section 2 presents the literature and hypothesis development. Section 3 describes the methodology used, while Section 4 the analysis and results. Finally, Section 5 presents the discussion and conclusions based in the results obtained, as well as the limitations and further research directions. 

## 2. Literature and Hypotheses Development

### 2.1. Corporate Environmental Responsibility and Readiness for Change

Corporate environmental responsibility (CER) represents the ecological dimension of corporate social responsibility, a concept used to describe companies that decide willingly, beyond the compliance with national legislations, to be active in the transition towards a better society and a sustainable environment [14,15]. CER implies that organizations are reconsidering the natural environment as a major stakeholder which can affect the consequences of their decisions and can also be affected by their actions [16,17]. Furthermore, the integration of CER into companies’ management is also the embodiment of the demand of green products from the stakeholder side. As so, the degradation of the environment has endorsed customers, governments and the public to be more attentive to environmental protection and engage in buying products from companies with such strategies and interests [18]. 

According to Weiner [19], as an organization-level construct, readiness for change refers to organizational members’ shared vision to carry out a certain change and their shared belief in the collective capability to implement that change. Organizational readiness for change varies depending on how much organizational members appreciate the change and how favorably they assess related task demands, resource availability, and situational factors. Specifically, change is expressed in an organization through the behaviors and work of individuals and it takes into account factors such as communication, coaching, leadership or organizational culture. Several strategies, tools, and techniques have been developed to address and manage change. Kerber and Buono [20] illustrates three key concepts: directed change (driven from above and based on authority and compliance), planned change (can occur from any point at the organizational level and seeks involvement and commitment), and guided change (emerges from people’s contributions and commitment to the organization’s goals). All of these changes are influenced by two factors: socio-technical uncertainty and business complexity. The transition towards a circular business implies a planned change as it must comply with national legislation and it requires involvement and commitment to the cause while shifting towards a circular model.

Previous empirical studies revealed the association between corporate social responsibility and organizational change [21], organizational commitment [22] and organizational effectiveness [23]. However, research investigating the relationship between CER and readiness for change towards a circular business model is missing. We argue that once CER is implemented in an organization it creates a suitable environment for the emergence of the readiness for change. While adopting a circular business model, the principles of environmental protection are regarded as strategic opportunities for organizational development and drivers for systematical seek of information to anticipate environmental changes.

**Hypothesis** **1.** *Corporate Environmental Responsibility is positively associated with Organizational Readiness for Change.*

### 2.2. The Mediating Role of Perceived CE Drivers

As they are confronted with new challenges related to the environment and sustainability, organizations must rely on internal and external drivers to innovate and create circular business models and implicitly “greener” business [24]. However, there is not just one important driver that guarantees a successful transition, but rather a fusion of facilitating factors, resulting from particular local conditions [25]. Since CE drivers are often conceptualized as a motivating factor for implementing the circular economy in an organization [26,27], they were introduced in our predictive model of readiness for change as a unifying element linking corporate environmental responsibility with readiness for change. Nevertheless, some researchers argue that the full comprehension of the general drivers for CE is a hard task due to the fragmentation of the field [28]. According to Govindan and Hasnagic [26] CE drivers can be grouped into different clusters such as: Policy and economy (laws concerning product take back and economy growth), Environmental protection, (the need to act on climate change, sustainable agriculture and the depletion of renewable resources), Society (the pressure of population growth particularly in urban areas, job creation potential and consumer awareness) and Product development (improving the resource efficiency of materials and energy use to increase the value of products).

**Hypothesis** **2.** *Perceived Circular Economy Drivers mediated the positive relationship between Corporate Environmental Responsibility and Readiness for Change.*

### 2.3. The Moderating Role of Perceived CE Barriers

The implementation of the circular economy in enterprises requires overcoming many barriers. A substantial amount of literature has been published on the barriers that companies face in implementing the circular economy [29]. The existence of organizational barriers is inherent since circular business models imply a drastic change. This new organizational mentality focuses not only on waste reduction by adopting “cradle-to-cradle” production models but also on the efficient use of resources, aiming at creating a harmonious relationship between society, economy and environment [30]. 

Govindan and Hasanagic [26] propose that these kind of barriers can be of internal or external nature, but regardless of their origin they both hinder organizational change [31]. External barriers refer to weak economic incentives that make it difficult for companies to implement CE [2,32], the lack of a standard system for performance indicators for CE measurement [18,33,34] or insufficient internalization of external costs as environmental costs are not taken into account [35]. On the other side, internal barriers refer, for example, to the increasing complexity of products that makes the recovery and reuse of products and components a massive challenge [36,37], major initial investment costs in the supply chain for CE implementation [38] or a higher priority given to other issues or requirements in the supply chain [37].

**Hypothesis** **3.** *In the mediated relationship between Corporate Environmental Responsibility and Readiness for Change, Perceived CE Barriers act as a moderator, such that is stronger for lower than for stronger levels of perceived CE Barriers.*

## 3. Methods

### 3.1. Sample and Data Collection

A total of 311 questionnaires from representatives of organizations and companies in Romania were collected via a Google Form survey from July to October 2020. The questionnaire was disseminated on social platforms such as Facebook or Linked-In. Survey was the method of choice as respondents could provide their systematic view of the perceived CE drivers to foster readiness of business to implement CE principles. The target audience was selected as a convenience sample, from the category of representatives of companies, businesses and organizations. The analyses were performed in SPSS and its PROCESS extension. In order to ensure validity and reliability, the Cronbach’s Alpha test was conducted, as it can be seen in the tables found in Section 4. 

The participants included 201 females (64.6%) and 100 males (24.9%). The average age of the sample was 29.77, with a standard deviation of 11.98. Of the 311 valid answers, 45.5% (141) of the respondents declared that they know what the concept of CE means and the rest of 54.5% (169) declared that they are not sure about what CE actually implies. In terms of their education, 52.1% have graduated high-school as the last form of education, 23.8% graduated university and 12.2% have graduated from a Master’s program. The rest of the respondents, 11.9% have graduated PhD, post-secondary school, gymnasium or other specializations. Most of the representatives of the organizations indicated that their company operates in the electrical and mechanical engineering industry (37.4%). The remaining organizations that make up the sample operate in the following sectors: Manufacturing (31%), Customer Relations (11%), Construction and Architecture (5.8%), Agriculture (5.2%), Media (3.5%), Education (3.5%) and Environmental Management (2.6%).

All participants gave their consent to participate in the study and allowed their data to be used for research purposes. Data collection was completely anonymous and voluntary. The questionnaire information was kept confidential.

### 3.2. Independent Variables

Corporate Environmental Responsibility was measured using a 6-item scale. The respondents were asked to rate on a 5-point Likert Scale, where 1 = “strongly disagree” and 5 = “strongly agree”, the degree to which the following statements were true concerning the principles of environmental protection. The indicators assessed were adapted from Li et al., 2019 are the following: 1. They are recognized by managers, 2. They are part of the organization’s strategy, 3. They are considered as a constraint to which they must adapt, 4. They are considered strategic opportunities for the development of our organization, 5. They push us to systematically seek information to anticipate environmental changes (risks, law, markets), 6. They are integrated into a highly engaged environmental policy with objectives, action plans, and procedures.

Perceived Circular Economy Drivers were measured using a scale consisting of 7 items. In this case as well, the respondents were asked to rate on a 5-point Likert Scale, where 1 = “strongly disagree” and 5 = “strongly agree” a series of affirmations about CE practices and principles in organizational context. The items were adapted from Govindan and Hasanagic 2018 and were: 1. They reduce environmental, social, community costs 2. They increase legitimacy 3. They develop new markets, 4. They diversify our products and services, 5. They improve our economic performance, 6. They raise the level of innovation, 7. They reduces the costs of risk and legal non-compliance. 

Perceived Circular Economy Barriers have been selected and adapted from Govindan and Hasanagic 2018 and were measured using a scale consisting of 11 items. The respondents were asked to rate on a 5-point Likert Scale, where 1 = “strongly disagree” and 5 = “strongly agree” on what degree the following statements about the implementation of CE describe the actual situation of their organization: 1. There is a lack of knowledge about circular products and practices 2. The tendency to replace rather than repair products exist in our organization, 3. There is a negative perception of reused/recycled content in new products, 4. There is a lack of maintenance and repair services, 5. Incorrect product design (not designed to be sustainable, easy to maintain, disassemble and reuse), 6. Non-integration in strategy, mission, vision, objectives and performance indicators, 7. Quality management processes and systems are organized in a linear manner, 8. Consumer culture and behavior; the price is No.1 in the purchase decision, 9. Lack of financial support, 10. Lack of adequate technology/infrastructure, 11. Lack of qualified professionals in environmental management.

### 3.3. Dependent Variable

**Figure 1:**
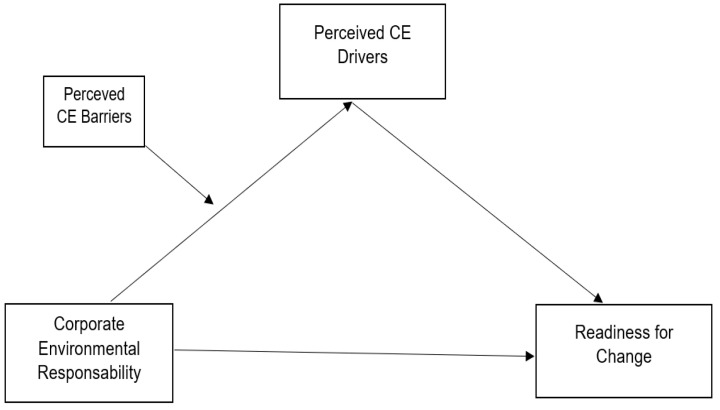
The moderated mediation model.

Readiness for change is the sole variable of the model (Figure 1) and it is measured with a scale consisting of 4 items on 5-Point Likert Scale, where 1 = “strongly disagree” and 5 = “strongly agree”. 

The items were: 1. My organization promotes the design of products for reuse, recycling, material recovery, components, 2. My organization encourages the creation of a recycling system for used and defective products, 3. My organization increases the amount of reused parts or recycled/renewable materials in the manufacturing process, 4. My organization increases purchase demand by reducing the cost of production and minimizing the impact of the product’s life cycle on the environment.

### 3.4. Control Variable

In order to reduce multicollinearity between the independent variables and to lower the influence of other variables that were not considered for this study, we used the experience that participants have in the field of environmental management. Years of experience could influence the manner in which organizations successfully deal with barriers while transitioning to a circular business model. 

## 4. Analyzes and Results

### 4.1. Descriptive and Correlational Analyzes

According to Table 1, sufficient reliability was demonstrated for all constructs through the Cronbach’s Alpha test, as all values are greater than the generally accepted benchmark of 0.70. This means that the items described in the previous section measure the same construct and show satisfactory inter-relatedness of the items within the test [39]. 

Table 2 shows the means, standard deviations and correlations of all variables in the study. As we expected, CER was positively correlated with readiness for change towards a circular business model (r = 0.450, *p* < 0.001). CER (r = −208, *p* < 0.001) and readiness for change (r = 0.216, *p* < 0.001) were negatively correlated with perceived CE barriers of the organizations. In addition, perceived CE drivers positively correlated with readiness for change (r = 608, *p* < 0.001) and CER (r = −0.369, *p* < 0.001).

### 4.2. Inferential Analyzes: Test of Moderated Mediation

The model was tested using the SPSS PROCESS extension (SPSS Inc., Chicago, IL, USA) and its model 7, designed by Andrew F. Hayes. The underlying method of such analysis is called Bootstrapping. It involves extracting a large number of samples from the raw data to calculate the confidence intervals for the indirect effect in this distribution. Therefore, the size of the indirect effect is no longer related to the normal distribution, but to a new distribution generated based on the effect observed in the samples generated by bootstrapping [40]. A number of 5000 resampled samples with a 95% confidence intervals were calculated for testing the theoretical hypothesis. If the 95% confidence interval did not include the 0 value, it meant that the statistics were significant and the hypothesis received empirical support. In what follows, the results obtained by applying this method of inferential analysis are described, according to the hypotheses formulated.

Firstly, the overall model explained 45% of the readiness for change variation (R = 0.456, F_(4, 305)_ = 20.16, *p* < 0.001). Table 3 presents all the main results. Hypothesis 1 receives empirical support, as there is a positive association between CER and readiness for change (B = 0.322, *t* = 5.58, *p* < 0.001).

As for Hypothesis 2, the positive predictive effect of CER on Readiness for change was significant as mentioned earlier, so the first condition for mediation validation was met. Moreover, the positive predictive effect of CER on Perceived CE drivers was significant (B = 0.639, *t* = 4.15, *p* < 0.001), while Perceived CE drivers had a significant positive predictive effect on Readiness for change (B = 0.569, *t* = 11.00, *p* < 0.001), thus the rest of conditions for validating the mediation were met. The 95% CI of the bias-corrected percentile bootstrap for the direct effect of CER on Readiness for change (B = 0.322, *t* = 5.58, *p* < 0.04) and the mediating effect of Perceived CE drivers did not include 0, indicating that the mediating effect was indeed significant. Therefore, hypothesis 2 is empirically supported.

The interaction term between CER and perceived CE barriers was significantly negatively related to readiness for change (B = −0.96, *t* = −2.014, *p* = 0.003), therefore hypothesis 3 receives empirical support. This means that perceived CE barriers act as a buffer that hinders the positive relationship between CER and readiness for change towards a circular business model. 

## 5. Conclusions

### 5.1. Conclusions

This study found that corporate environmental responsibility is positively related to readiness for change towards a circular business model. The validation of moderated mediation model proves that perceived CE drivers act as mediators in this relationship. Perceived CE barriers moderate the relationship between corporate environmental responsibility and readiness for change-acting like a buffer diminishing the positive effect. The discussion of circular business models is more relevant today than ever, as stakeholders around the world pursue stringent goals related to climate change and the decoupling of human well-being from the irrational linear consumption model of finite resources. CE represents the solution that combines economic development with environmental sustainability by creating sustainable business models based on the extension of the product life cycle and the integration of companies with external partners to share services, tangible products, urban industrial symbioses, etc. [41,42]. However, there are still challenges and risks associated with implementing a circular economy model which are related to the framework of the CE model, which is inherently interconnected—the idea of closing material loops does not only concern a single company and its boundaries, but requires the participation of a system of business models that act together [43]. 

As so in this context, readiness for change is imperative in the business management in order to enhance the power of drivers towards adopting circular economy principles and diminish the hindering effect of barriers. 

As the research paper under-discussion has found out, Corporate Environmental Responsibility is positively influencing the Readiness for Change in organizations. Meaning that adopting CER as part of the management of business can build on resilience of the company in the face of new challenges and requests from national and European laws and regulations with regard to CE. These findings are also supported by Li et al. [14] and Belal et al., [44].

On another hand, Perceived CE Drivers by the respondents positively mediate the relationship between CER and readiness for change. Thus, they can act as facilitators in the relationship between CER and Readiness for Change to enhance the transition towards circular economy. These findings are also supported in scientific literature, in Doran and Ryan [45], Quarshie et al. [46], and Eijik [47]. 

Perceived CE barriers by the respondents of the study moderate the relationship between CER and Readiness for Change such as, the relationship is stronger for lower levels of CE barriers identified. Therefore, the higher the barriers identified, the lower the relationship between CER and Readiness for Change. This hypothesis confirmed through the current research paper is also visible in Eijik, [47] and Govindan and Hasanagic [26].

The study not only provides a perspective of circular business models on revealing the pathway between CER and readiness for change to a circular model but also complements previous researches that linked corporate social responsibility with organizational effectiveness and conceptualized it as a tool for creating future environmental protection [48,49]. 

More importantly, our findings can contribute to the elaboration of a conceptual framework for embedding circular economy in the corporate social responsibility strategies of organization. As complexity and turbulence of the environment is increasing nowadays more than ever, it becomes vital that companies develop competitive management models aimed to generate profits while meeting the expectations of society. Such demands include a sustainable long-term view and the adoption of circular business models as part of their management strategies [50]. In addition, by incorporating the CE in organizational strategies targeting social responsibility, organizations can overcome one of the major critiques of the circular paradigm—its silence regarding the social dimension, and excessive focus on the redesign of manufacturing and service systems that benefit the bio-sphere. While the reduction of the use of finite resources clearly brings benefits globally, there is no explicit acknowledgment of the social aspects that are present in the conceptualizations of circular development [51,52,53].

Therefore, organizations could develop strategies that incorporate the organizational barriers and drivers for eco-innovation and achieve organizational changes that lead them to incorporate the circular economy. Moreover, we need to be aware that although several findings suggest clear benefits for companies adopting circular business models, in practice there may be several other barriers, such as difficulties in assessing future benefits compared to current costs, knowledge needs, and market pull and push factors, such as technology availability and consumer demand for green products [31]. 

### 5.2. Limits and Future Research Directions

The present study has some limitations that must be taken into account when interpreting the results obtained. Given the sample size, the results of the study cannot be extrapolated to develop general assumptions on a larger scale. For the results to be representative at a national level, a larger number of respondents with a greater diversity of backgrounds is required. For example, the size of the company or its financial resources may influence its willingness to switch to a circular economy business model.

The cross-sectional correlation design implies that measurements are made only once. Therefore, explanations for the dynamic nature of the variables cannot be generated or causality otherwise inferred.

Another limitation of this type of design is the fact that the data were collected using an online questionnaire, which meant that we had no control over the environmental conditions that could interfere with the accuracy of the answers, altering those in follow the results. Lastly, the data was based on self-report measures, which may lead to a high frequency of desirable answers.

Therefore, further longitudinal studies need to be conducted to validate the moderated mediation model. Since this study focused on organizations in general, further studies focusing on specific industries need to be conducted.

## Figures and Tables

**Table 1:** Reliability Analysis Results.

Variable	Mean	N of Items	Cronbach’s Alpha
Corporate Environmental Responsibility	3.35	6	0.818
Readiness for change	3.23	4	0.924
Perceived CE Drivers	3.23	7	0.941
Perceived CE Barriers	3.22	11	0.950

**Table 2:** Descriptive statistics and correlations for continuous variables.

Variable	N	M	SD	1	2	3	4	5
1. Corporate Environmental Responsibility	311	3.34	0.818	1	.			
2. Readiness for change	311	3.23	1.01	0.45 **	1			
3. Perceived CE Drivers	311	3.23	0.913	0.369 **	0.608 **	1		
4. Perceived CE Barriers	311	3.22	1.01	−208 **	−0.216 **	0.321 **	1	
5. Control Variable	311	1.66	0.82	−0.065 **	−0.086	−0.054	−0.119 *	1

** *p* < 0.01. * *p* < 0.05.

**Table 3:** The Moderated Mediation Analysis.

Predictors	B	SE	*t*	*p*	LLCI	ULCI
Outcome variable: Readiness for change						
Corporate Environmental Responsibility	0.322	0.057	5.58	0.00	0.2091	0.4366
Perceived CE Drivers	0.569	0.051	11.00	0.00	0.4674	0.6709
Corporate Environmental Responsibility -> Readiness for change (direct effect)	0.322	0.057	5.58	0.00	0.2091	0.4366
Outcome variable: Perceived CE Drivers						
Corporate Environmental responsibility	0.639	0.153	4.15	0.00	0.3367	0.9422
Perceived CE Barriers	0.541	0.162	3.34	0.09	0.2228	0.8609
CER x Perceived CE Barriers (interaction effect)	−0.96	0.048	−2.014	0.04	−0.195	−0.0023

Notes: B (beta), regression coefficient; SE, standard error; *t*, *t*-test value; LLCI—lower limit of confidence interval, ULCI—upper limit of confidence interval.
